# Predictors of the regional variation of prostatectomy or radiotherapy: evidence from German cancer registries

**DOI:** 10.1007/s00432-020-03140-x

**Published:** 2020-03-04

**Authors:** Daniel Medenwald, Julia Ferencz, Dirk Vordermark

**Affiliations:** 1 Department for Radiation Oncology, Martin-Luther University Halle-Wittenberg, Halle, Germany; 2 OnkoZert, Independent Certification Institute of the German Cancer Society, Neu-Ulm, Germany

**Keywords:** Prostate cancer, Radiotherapy, Surgery, Germany, Health service research, Certified cancer centers, Regional variation

## Abstract

**Objective:**

To assess the association of public health parameters with the regional variation in the initial treatment for prostate cancer.

**Methods:**

We used data from German epidemiologic cancer registries for the years 2009–2013. Presence of a certified cancer center, a radiotherapy and/or urology institution, the district-specific GDP, and population density were used as predictors. Patients with indication for adjuvant treatment were excluded (T3b). Only districts with defined quality criteria were eligible. We used general linear mixed models (equivalent to logistic regression) with a covariance matrix weighted by the Euclidean distances between districts. Models were adjusted for age, grading, and TNM stage. We performed sensitivity analyses by imputing missing data with multiple imputation and considering extreme case scenarios. We applied inverse probability weighting to account for missing values.

**Results:**

When radiotherapy/surgery is compared to neither treatment, the probability for the latter was higher in East than in West Germany (OR 1.7, 95% CI 1.43–2.02). The same was true for districts with both, a radiotherapy and urologic treatment facility (OR 1.43, 1.19–1.72). Analyzing radiotherapy vs. surgery, the probability for prostatectomy was inversely associated with the presence of a radiotherapy unit when compared to districts with neither treatment facility (OR 0.52, 95% CI 0.38–0.73). Patients treated in East Germany were more likely to receive a surgical treatment (OR 1.34, 95% CI 1.08–1.66). Sensitivity analyses revealed no relevant change of effect estimates.

**Conclusion:**

Treatment differs between East and West Germany and is associated with the presence of a radiotherapy or urology clinic.

**Electronic supplementary material:**

The online version of this article (10.1007/s00432-020-03140-x) contains supplementary material, which is available to authorized users.

## Introduction

Curative treatment of prostate cancer remains controversial building on the alternative options of prostatectomy or radiotherapy. Randomized trials found a comparable efficacy of treatments in terms of overall survival, while patterns of adverse events differ between both options (Hamdy et al. 2016). Respecting these findings, the preference of the informed patient should guide the choice of treatment.

For certain patient cohorts, ‘active surveillance’ and ‘watchful waiting’ are further options to target prostate cancer. The former is restricted to patients with a very low risk and entails an active patient monitoring. In contrast, ‘watchful waiting’ is a passive approach, which abstains from either an interventional treatment or ‘active surveillance’, but would only consider treatment when the disease becomes symptomatic. Patients with a restricted life expectancy are candidates for this approach.

From a public health perspective, treatment environments differ in terms of the availability and established practice in the administered treatment. Externalities ranging from the neighborhood economic status to the availability of treatment units might affect preferences of physicians and patients as it was found in a recent population-based study (Tomic et al. 2018). Such effects might ultimately lead to substantial differences in terms of survival between regions within a country (Skyrud et al. 2016). While external factors explain survival differences, it remains unclear how treatment preferences differ in the first place independently from such externalities. This is especially relevant in entities where there are treatment options with comparable outcomes as it is the case for prostate cancer (see above).

German population-based, that is epidemiological, cancer registries cover almost all incident cancer cases among residents of their respective jurisdictions. They offer a unique possibility to compare regions in terms of the administered treatment on a population-based level (Schubert-Fritschle et al. 2017). It is the objective of this study to identify treatment preferences in a population-based sample.

## Methods

### Data set

We used data from the epidemiological cancer registries, as they are made available by the German Centre for Cancer Registry Data (Zentrum für Krebsregisterdaten)—in accordance with the Federal Cancer Registry Data Act (Bundeskrebsregistergesetz)—to the third parties on application. The observational period encompassed the years from 2009 to 2013. The data set covers incident cancer cases in Germany and provides information on time of diagnosis, cause and date of death, histopathological grading, TNM stage, place of residence of the patient (district level), and age at the time of diagnosis.

Information on the regional presence of certified organ cancer centers in districts were obtained from the Deutsche Krebsgesellschaft (DKG, German Cancer Society through OnkoZert, the Independent Certification Institute of the German Cancer Society) on a year-specific basis for the years of diagnosis between 2009 and 2013. The institute supervises and conducts the process of certification in Germany.

Data on radiotherapy institutions (in- and outpatient) and clinics for urology (inpatient only) are based on the ‘Deutsches Krankenhausverzeichnis’ (German hospital directory) as it is published by the Federal Statistical Offices (Statistische Ämter des Bundes und der Länder 2014) and information provided by the ‘Kassenärztliche Bundesvereinigung’ (the National Association of Statutory Health Insurance Physicians), respectively.

Data on the geographic parameters such as number of inhabitants, area size, and district types were obtained from the ‘Bundesinstitut für Bau-, Stadt- und Raumforschung’ (https://www.bbsr.bund.de/) and the ‘Federal Statistical Office’, respectively (Statistische Landesamt Baden-Württemberg 2016).

### Inclusion and exclusion criteria

To reduce possible biases related to selective reporting, we first conducted a hierarchical inclusion process aimed at taking only data from regions with a sufficient quality of recording into account. Second, we adjusted statistical models to account for missing data as described below.

**Fig. 1:**
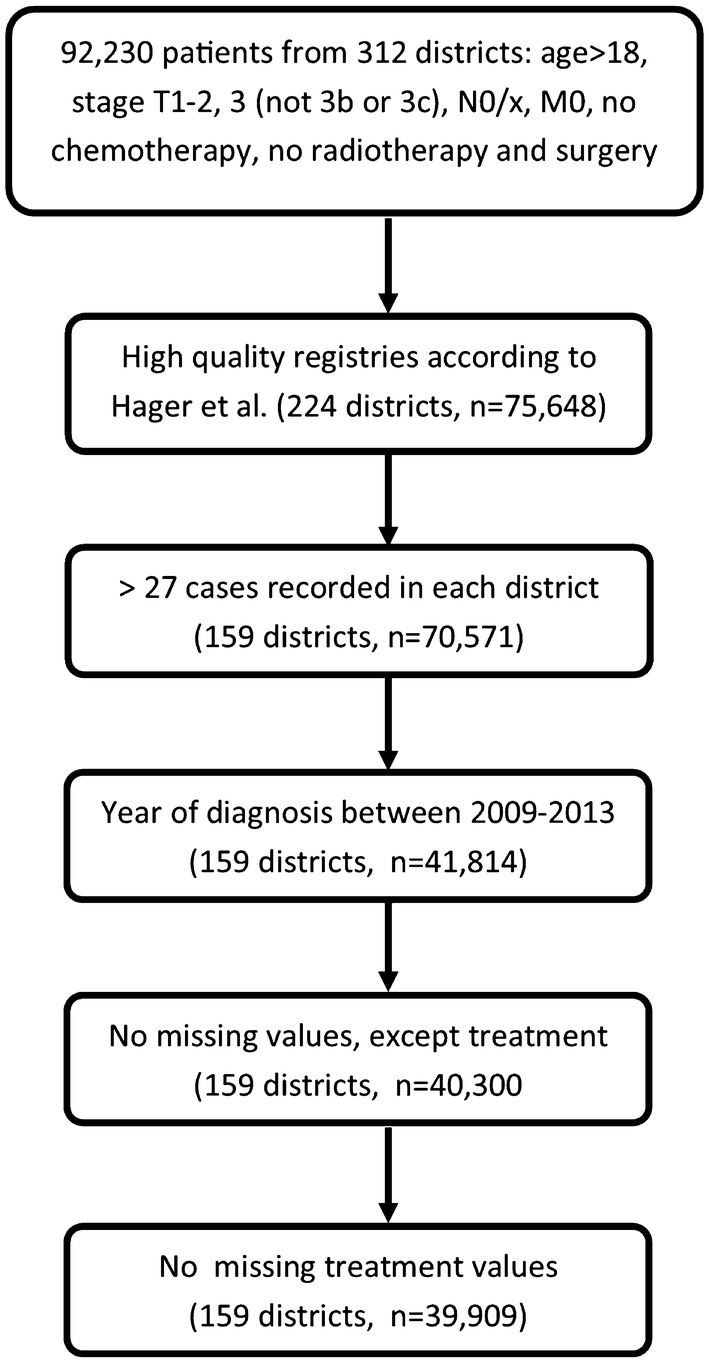
Hierarchical inclusion and exclusion process on patient and district levels

Figure 1 depicts the inclusion process. After the identification of clinical cases where no adjuvant treatment is advised by German S3 guidelines (version 5.0, exclusion of cases with TNM of T3b or higher as recorded, and N+; only cases without metastases, M0), we additionally excluded patients that received chemotherapy, or both, radiotherapy and surgery. Cases with a T stage of T3b were as excluded as here seminal vesical involvement warrants the indication for adjuvant radiotherapy. To ensure a sufficient data quality, we only considered cases from federal registries meeting the completeness criteria according to Hager et al. (2015).

Based on these selected cases, to estimate the proportion of applied treatments with a sufficient precision, we defined the minimum case number within districts as follows: the proportion of cases treated by means on radiotherapy was 11.8% in the whole data set. Subsequently, we presumed the precision of a 90% confidence interval (CI) to be 10% around this estimate (Julious 2009). This leads to a number of at least 28 cases recorded in a district for it to be considered for further analyses. Due to the availability of information on certified cancer centers between 2009 and 2013, cases diagnosed in this period were eligible.

In our study, the term ‘no active treatment’ refers to patients that received neither radiotherapy nor surgery.

### Statistical analyses

To take spatial autocorrelations between districts into account, we used general linear mixed models (PROC GLIMMIX in SAS) with a covariance matrix weighted by the Euclidean distances between districts. Here, we computed odds ratios with 95% confidence intervals from a linear model with a logit link function and a binomial distribution of residuals.

Models were adjusted based on two levels including an individual patient level for age, age squared, propensity of treatment in relation to age (the latter two to account for non-linearity), grading, and TNM stage. On a second district level, the parameters of presence of a certified cancer center or a radiologic/urologic institution, gross domestic product, inhabitants per square kilometer, district size, and larger region of East and West Germany were included as covariates. We standardized metric values by dividing by the standard deviation to make estimate comparable between parameters.

To adjust for missing values in the model, we inversely weighted observations by the estimated probability of finding a complete case in the respective district. To predict this probability, we used a logistic regression model including the predictors of year of diagnosis, age and their interactions, federal state, presence of certified cancer center, radiologic institution, or urologic clinic, respectively. Thus, presuming missing values to occur at random, after adjusting for the respective variables, we are able to correct for a bias caused by missing data when the criterion of recoverability is applied.

### Sensitivity analyses

We performed an analysis where missing values were imputed by means of multiple imputation (fully conditional specification for categorical variables) (van Buuren 2007). This approach uses evidence from the existing data to generate results that would have occurred had there been no missing data. This is true if the assumption of missing at random (MAR) holds. That is, adapted to the present study, missing values are solely generated by observed variables such as the presence or absence of a therapeutic institution in a district.

As there were missing treatment variables in some districts (restricted to West Germany; Figure S1 in the supplement shows the proportion of missing cases), we repeated the analyses described above in an extreme case scenario where all missing values were set to either radiotherapy or surgery.

We performed a further sensitivity analysis where we excluded all cases from Berlin and Saxony-Anhalt as the incidence reported in those states was considerably low (Koch-Institut R 2015). This might indicate a higher proportion of incident cases that failed to enter the registry.

Apart from missing data on treatment, subjects might not have entered the registry in the first place. To account for this level of missing data, we again presumed an extreme case scenario. Here, we used the coverage of cancer registries as estimated by the Robert-Koch-Institute (Stefan Hentschel 2008). Effect estimates where again adjusted for an extreme case scenario where the proportion of cases not recorded in the registries were all set to radiotherapy or surgery treatment. Finally, 40,300 cases fulfilled the inclusion criteria for sensitivity analyses and 39,909 for complete case analyses. For statistical analyses, we used SAS 9.4.

## Results

### Basic characteristics

**Fig. 2:**
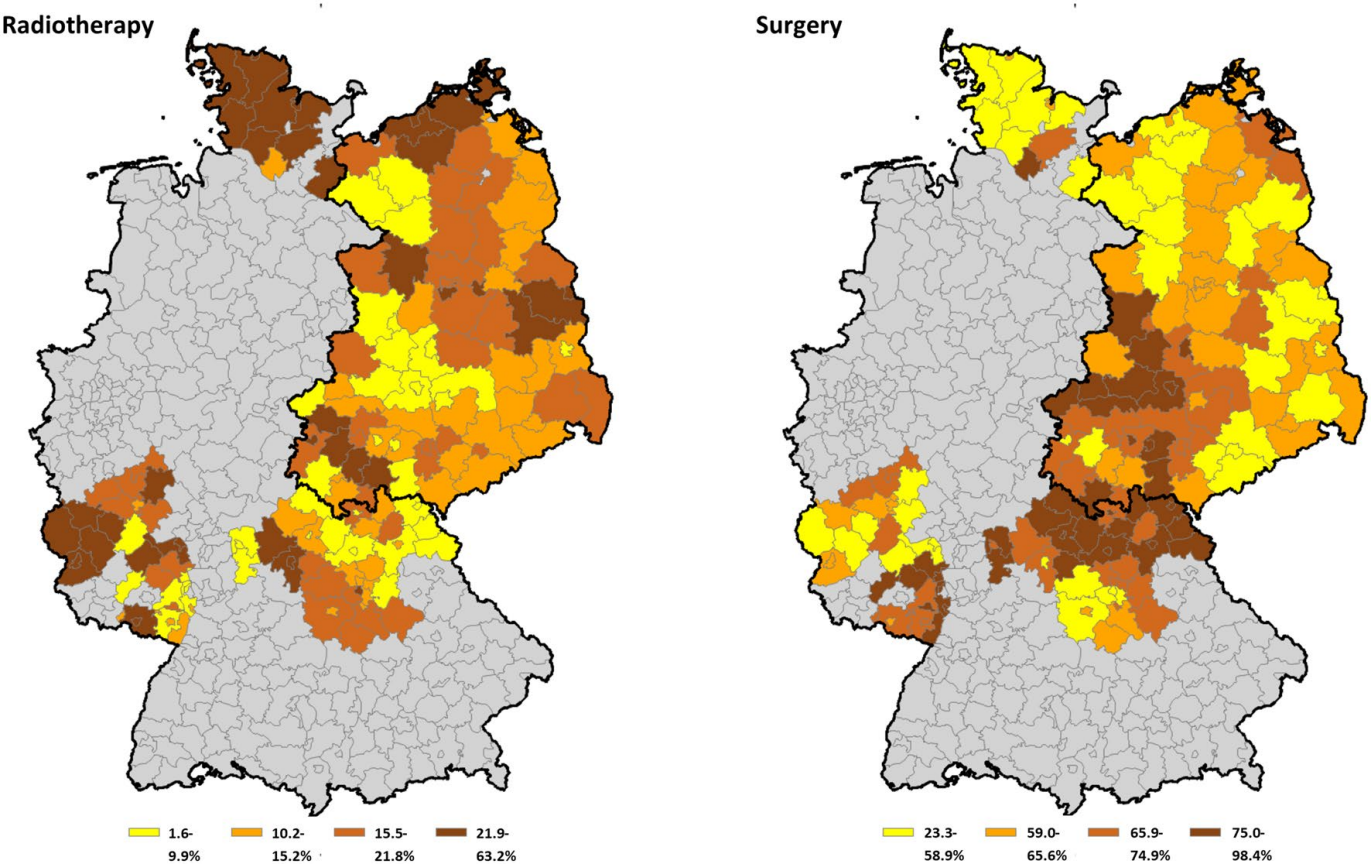
District-specific proportion of cases treated with radiotherapy and surgery from all cases. Possible treatments were radiotherapy, surgery, or neither treatment (displayed as proportions)

As Fig. 2 indicates, surgery is the standard treatment in central Germany with a proportion exceeding 75% of incident cases. The proportion of patients receiving radiotherapy was higher in Schleswig–Holstein when compared to other states.

### Predicting treatment

**Fig. 3:**
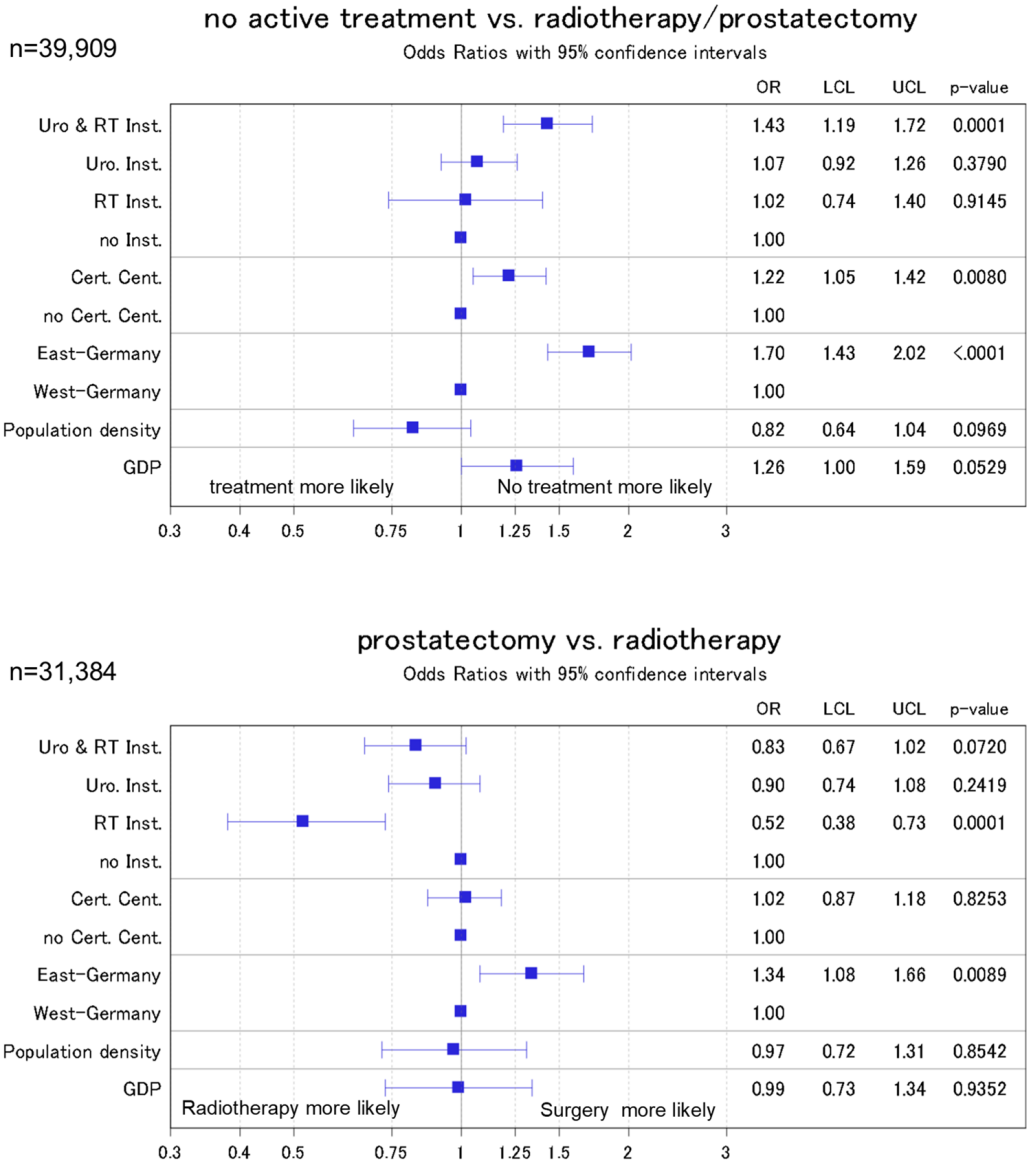
Odds ratio from logistic regression models of the association of public health parameters with the administered treatment. Models were adjusted for patient characteristics (age, age squared, probability of treatment in relation to age, grading, and TNM stage) with inverse probability weighting to account for missing data. ‘Radiotherapy institutions’ refers to inpatient and outpatient institutions; ‘urologic institutions’ refers to inpatient units only

When ‘neither treatment’ is compared to radiotherapy or surgery, we found that the probability for the former was higher in East than in West German registries (OR 1.7, 95% CI 1.43–2.02, Fig. 3). The same was true for districts with a radiotherapy and urologic treatment facility when compared to districts where neither is present (OR 1.43, 1.19–1.72) in models adjusted for patient characteristics.

In the analysis of radiotherapy vs. surgery, we found that the probability for treatment by prostatectomy was inversely associated with the presence of a radiotherapy unit in a respective district when compared to districts with no treatment facility, neither radiotherapy nor urology (OR 0.52, 95% CI 0.38–0.73). When compared to West Germany, patients living in East Germany were more likely to receive a surgical treatment (OR 1.34, 95% CI 1.08–1.66).

### Sensitivity analyses

**Fig. 4:**
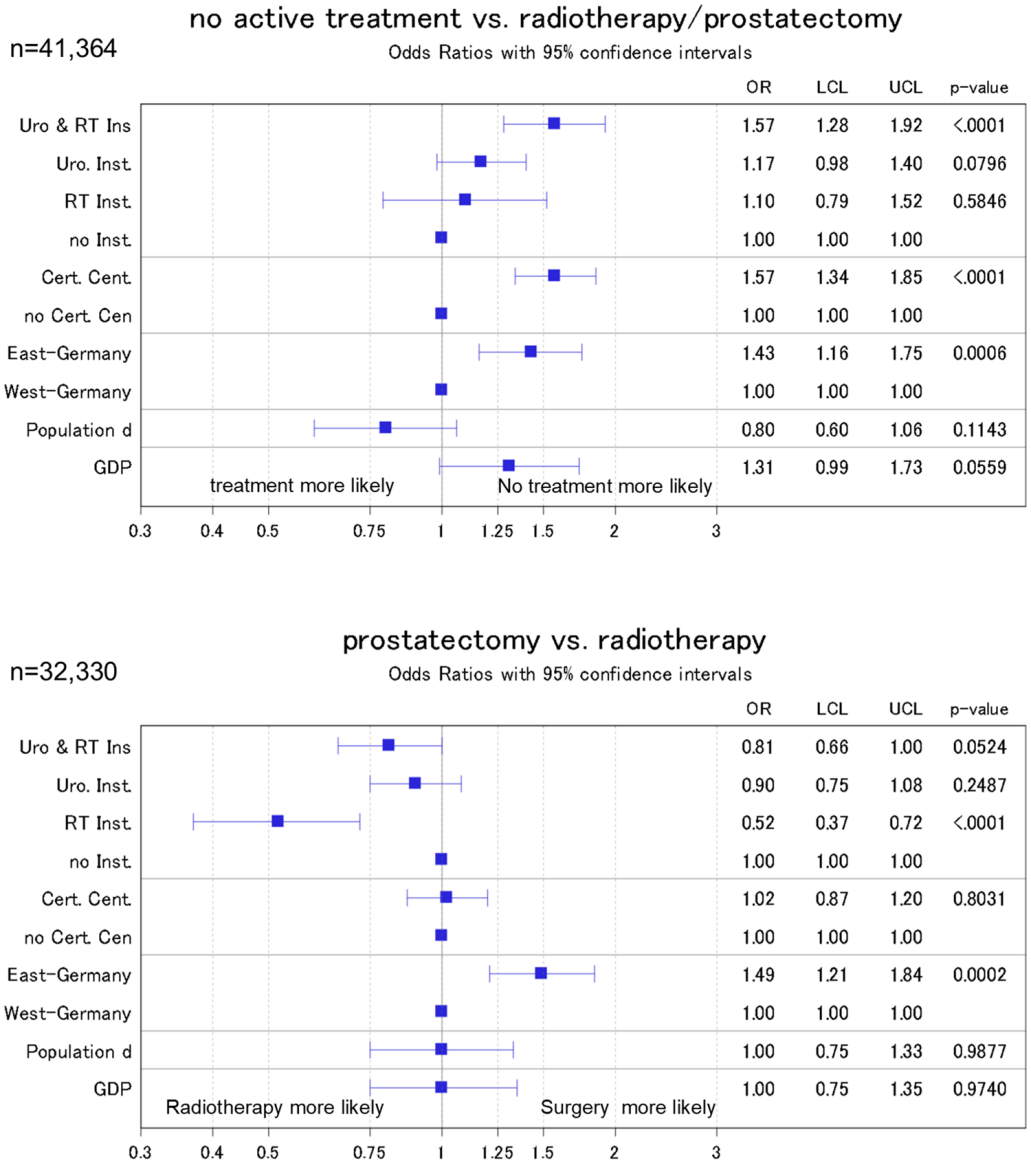
Odds ratio from logistic regression models of the association of public health parameters with the administered treatment. Sensitivity analysis where all missing values were imputed by means of a multiple imputation procedure. Models were adjusted for patient characteristics (age, age squared, probability of treatment in relation to age, grading, and TNM stage) with inverse probability weighting to account for missing data. ‘Radiotherapy institutions’ refers to inpatient and outpatient institutions; ‘urologic institutions’ refers to inpatient units only

When we applied multiple imputation to account for missing data, we found minor changes in the observed effect estimates. In detail, effect estimates increased in most estimations apart from the effect between East and West Germany (institutional effect from 1.43 to 1.57; cancer center effect from 1.22 to 1.57; effect of East/West Germany from 1.7 to 1.43, Fig. 4).

Predicting neither treatment compared to surgery or radiotherapy (Figure S2), we found that the effect estimates of the comparison of East and West Germany changed to 1.73 when missing values were regarded as having been treated with radiotherapy or surgery, and 1.17 when they were set to ‘neither treatment’. The change in the institution effect was minor (OR 1.31 for the former scenario or 1.27 for the latter, respectively).

Comparing the probability for prostatectomy vs. surgery in a sensitivity analysis where we set all missing treatment data to surgery (Figure S3), we found a weaker effect between East and West Germany (OR 1.24, 95% CI 1.02–1.51), while the effect of the presence of a radiotherapy unit remained virtually unchanged (OR 0.53). In the opposite case (all missing set to radiotherapy), the effect between East and West Germany increased considerably to an OR of 1.67.

When we excluded cases from Berlin and Saxony-Anhalt, we found minor alterations in the effect estimates, but an increase in the width of the confidence interval for GDP (Figure S4 in the supplement).

Analyzing the potential bias due to an incomplete coverage of incident cases by cancer registries, we assumed the average coverage to be 85%. Thus, this might account for a maximum odds ratio of 1/0.85/0.85 = 1.38, which is not sufficient to fully negate the effect observed between East and West Germany in terms of the application of neither treatment where we found an OR of 1.7.

## Discussion

In summary, we found that no active treatment, applying neither radiotherapy nor surgery, is more favored in East than in West Germany. Likewise, patients living in districts with both, a radiotherapy and urology treatment unit, or with a certified cancer center are more likely to receive no active treatment. Furthermore, patients are especially likely to receive prostatectomy compared to radiotherapy when they live in a district where only a radiotherapy institution is present or in East Germany.

To our knowledge, this is the first study that examines treatment preference in East and West Germany based on a population-based sample focusing on prostate cancer. A population-based approach entails the advantage of a nationwide picture that is not limited by data that might themselves affect treatment choices such as data from health care insurances or health care providers.

Coming to the clinical consequences of our findings, the identification of factors that lead to treatment clusters becomes a key objective of health care research. In a randomized trial (randomized between usual care and additional information from a decision aid), there was evidence that the visited hospital was a predictor for treatment, but not age and tumor characteristics (van Tol-Geerdink et al. 2013). These findings are well in line with our results where hospitals as they are part of a larger regional framework of East and West Germany were associated with treatment choice.

Treatment preferences are a complex interplay between individual patient characteristics and external factors such as the socioeconomic characteristics across areas. On a patient level, further studies found that factors such as risk attitudes (López-Pérez et al. 2017), age, or tumor stage were related to the preferred treatment (Sommers et al. 2008). Another study in a collective of 11,892 men with localized prostate cancer found a strong inter-institutional treatment variation in treatment preference ranging, e.g., for external radiotherapy, from 2 to 33% (Cooperberg et al. 2010). This differences could not be explained by case-mix variability or known patient factors, while institutional practice sites accounted for these variations to a varying degree (13% for androgen deprivation monotherapy; 74% for cryoablation) (Cooperberg et al. 2010).

An analysis based on SEER (Surveillance, Epidemiology, and End Results) data addressed treatment patterns in patients above 66 years diagnosed with T3 and T4 prostate cancer (Lowrance et al. 2012). Here, the authors reported an increase in multimodal therapy with radiotherapy and androgen deprivation therapy (ADT) between 1998 and 2005. Only a minority of 15% of the patients received no active treatment, while 36% received ADT only. As ADT is dependent on the application of radiotherapy, we abstained from accounting for ADT in statistical models in order to avoid bias from strongly correlated variables and collider bias (bias resulting from conditioning on variables that are dependent on two parent variables, e.g., radiotherapy and cancer stage for ADT in our case).

Returning to treatment outcomes in Germany, we found previously that people living in East Germany have a comparable mortality from prostate cancer as their West German counter-parts (Medenwald et al. 2017). Mortality rates in East and West Germany aligned only during the last 2 decades after the year 2000. Thus, the difference in treatment pattern seems not to result in a measurable difference in mortality. However, because of the favorable survival prospect of patients suffering from prostate cancer in an early stage screening programs might affect mortality from prostate cancer most strongly (Schröder et al. 2014).

Germany is characterized by a high rate of patients treated with surgery when compared to. e.g., USA (Hager et al. 2015). Hager et al. computed a rate of prostatectomy of 36.1% in the USA and 66.2% in Germany, while the rates of radiotherapy were 38.4% and 11.8%, respectively. Again data from the US-American and German registries showed an increased utilization of prostatectomy in locally advanced cases of prostate cancer (Hager et al. 2017). However, in both studies, the authors did not differentiate between East and West Germany and did not account for differences in the age composition and in economic factors. In our data, shifting only slightly from the preference for prostatectomy, radiotherapy was more likely when there was a radiotherapy unit but no urologic institution.

As our results emanate from multivariate adjusted models, causes leading to the mentioned treatment choices descend from independent origins. The finding that an active treatment is less likely in districts that are equipped with both, a radiotherapy and urology institution, or a certified cancer centers might reflect a better infrastructure for ‘active surveillance’ or ‘watchful waiting’. Still, other causes might underlie the higher likelihood for no active treatment in East Germany. As ‘active surveillance’ is a feasible treatment in a selective group of patients with an early stage, regions with a high density of treatment units might offer a better screening leading to a higher frequency of early cases suitable for ‘active surveillance’. Although, such an effect is unlikely as we adjusted for stage, there are parameters in the definition of early suitable cases, such as the degree of punch biopsy, which our data fail to record. This reasoning needs further investigation by prospective studies that, however, have the disadvantage of not covering the entire population.

### Limitations

In our study, the strongest source of bias might originate from missing data that are not missing at random. That is, after observed confounders of the recording process have been taken into account, missing values are still not random but are associated with unobserved parameters. However, such biases are unlikely to affect our findings because of two reasons.

As the sensitivity analyses revealed, our findings were robust even when we assumed extreme case scenarios. The only effect estimate that showed some variation between considered scenarios was the comparison of East and West Germany, while the general conclusion of a stronger preference of surgery in East than in West Germany remained unchanged. In the analysis where we imputed missing values by means of multiple imputation rather than the aforementioned extreme case scenarios, we found little change in the prescribed estimates. This underlines again the robustness of our findings against biases resulting from incomplete recordings.

Because clinical institutions rather than the individual patient conduct the recording of cases, the assumption that the data are ‘missing at random’ after covariate adjustment is well founded. In other words, only observed variables generate missing values. In multivariate models, we adjusted for these parameters, and thus, we could estimate results without biases from processes causing missing data. Likewise, we can presume that after we adjusted for public health care parameters, the selection process (driven by the same parameters) and the outcome are independent leading to a recoverability of (causal) relations (Pearl and Bareinboim 2014).

A large study in English lung cancer patients showed that the variation in treatment choice is associated with survival (Møller et al. 2018). The authors stated that deaths could have been avoided had active treatment more consistently applied. However, in our study of prostate cancer patients, such analyses are difficult to perform due to a longer and better survival time in this entity. Thus, we cannot estimate the survival potential survival effect.

As values were missing in West German registries, computed estimates may be different had there been no missing values. However, as the conservative sensitivity analyses of complete case scenarios revealed, missing values are unlikely to change the general conclusions of this study.

## Conclusion

In summary, we found that the administered treatment of prostate cancer varies systematically between East and West Germany according to the availability of radiotherapy institutions.

## Electronic supplementary material

Below is the link to the electronic supplementary material.

Figure S1: Percentage of missing values in considered districts (TIFF 784 kb)

Figure S2: Odds ratio from logistic regression models of the association of public health parameters with the administered treatment for the comparison of ‘neither treatment’ with radiotherapy/surgery. Sensitivity analysis where all missing values were presumed to have been treated with ‘neither treatment’ or radiotherapy/surgery, respectively. Models were adjusted for patient characteristics (age, age squared, probability of treatment in relation to age, grading and TNM-stage) with inverse probability weighting to account for missing data. ‘Radiotherapy institutions’ refers to in- and outpatient institutions, ‘urologic institutions’ refers to inpatient units only. (TIFF 454 kb)

Figure S3: Odds ratio from logistic regression models of the association of public health parameters with the administered treatment for the comparison of prostatectomy with surgery. Sensitivity analysis where all missing values were presumed to have been treated with surgery or radiotherapy, respectively. Models were adjusted for patient characteristics (age, age squared, probability of treatment in relation to age, grading and TNM-stage) with inverse probability weighting to account for missing data. ‘Radiotherapy institutions’ refers to in- and outpatient institutions, ‘urologic institutions’ refers to inpatient units only. (TIFF 437 kb)

Figure S4: Odds ratio from logistic regression models of the association of public health parameters with the administered treatment. Sensitivity analysis where cases from Saxony-Anhalt and Berlin were excluded. Models were adjusted for patient characteristics (age, age squared, probability of treatment in relation to age, grading and TNM-stage) with inverse probability weighting to account for missing data. ‘Radiotherapy institutions’ refers to in- and outpatient institutions, ‘urologic institutions’ refers to inpatient units only. (TIFF 454 kb)

## Abbreviations

ADT: Androgen deprivation therapy
Cert. Cent.: Certified center
CI: Confidence interval
GDP: Gross domestic product
Inst.: Institution
MAR: Missing at random
OR: Odd ratio
RT: Radiotherapy
SAS: Statistical analysis system
SEER: Surveillance, epidemiology, and end results
Uro.: Urology
