# Supplementary figures and images for: Predictors of the regional variation of prostatectomy or radiotherapy: evidence from German cancer registries
Source: J Cancer Res Clin Oncol. 2020 Mar 4;146(5):1197–204. doi: 10.1007/s00432-020-03140-x (PMC7142037; doi:10.1007/s00432-020-03140-x)

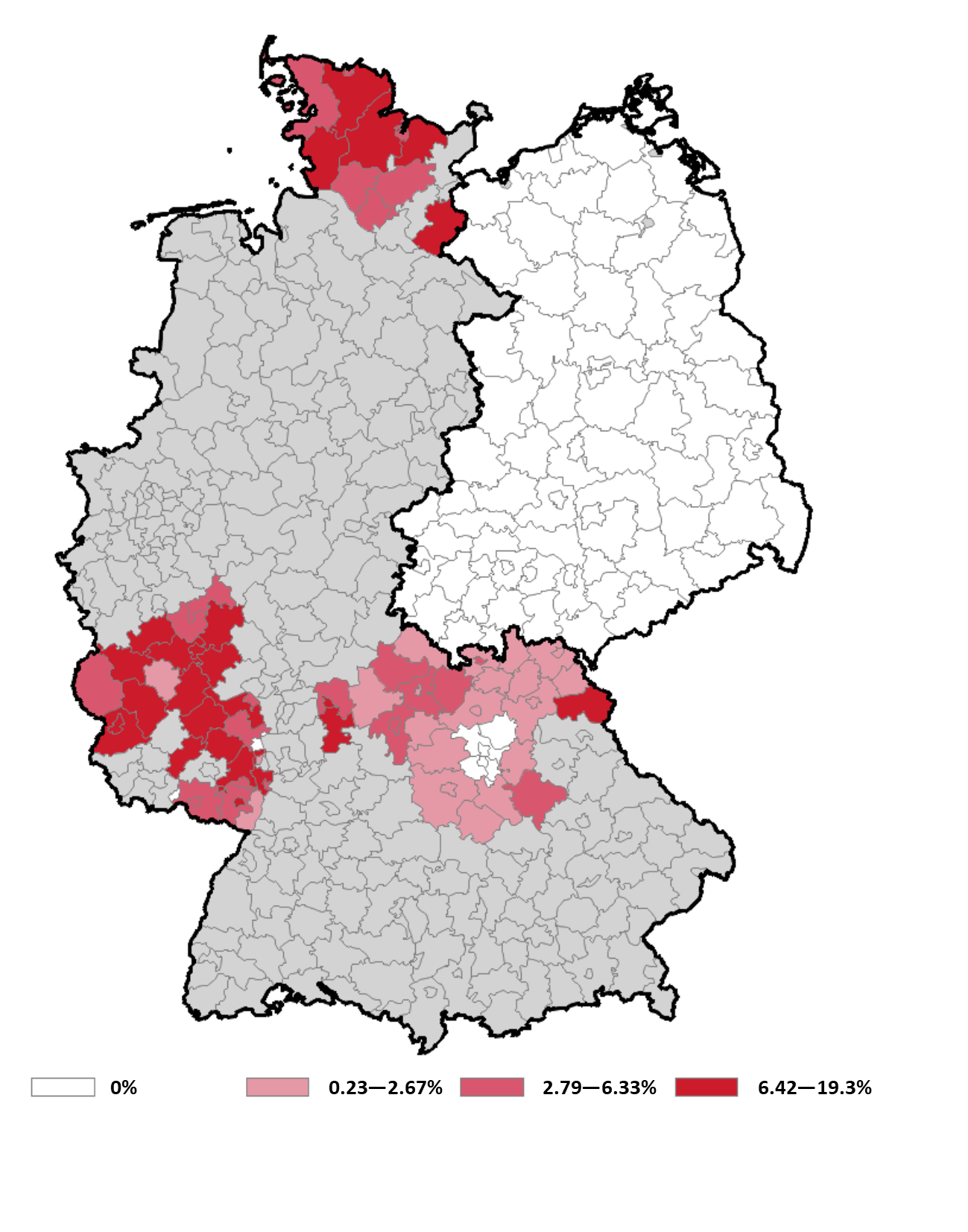

Supplement: Supplementary file 1 — Figure S1: Percentage of missing values in considered districts (TIFF 784 kb) [file 432_2020_3140_MOESM1_ESM.tif]

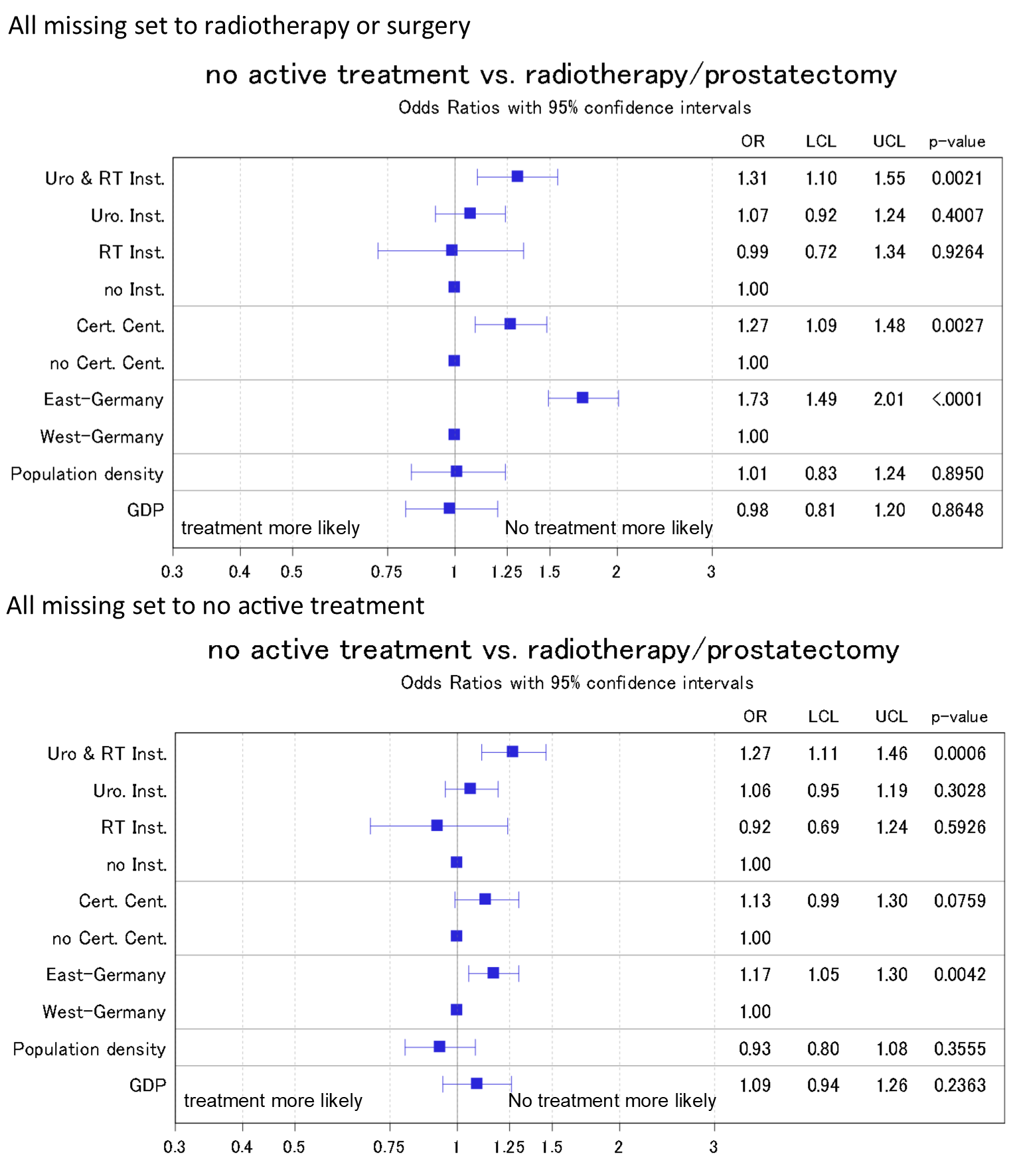

Supplement: Supplementary file 2 — Figure S2: Odds ratio from logistic regression models of the association of public health parameters with the administered treatment for the comparison of ‘neither treatment’ with radiotherapy/surgery. Sensitivity analysis where all missing values were presumed to have been treated with ‘neither treatment’ or radiotherapy/surgery, respectively. Models were adjusted for patient characteristics (age, age squared, probability of treatment in relation to age, grading and TNM-stage) with inverse probability weighting to account for missing data. ‘Radiotherapy institutions’ refers to in- and outpatient institutions, ‘urologic institutions’ refers to inpatient units only. (TIFF 454 kb) [file 432_2020_3140_MOESM2_ESM.tif]

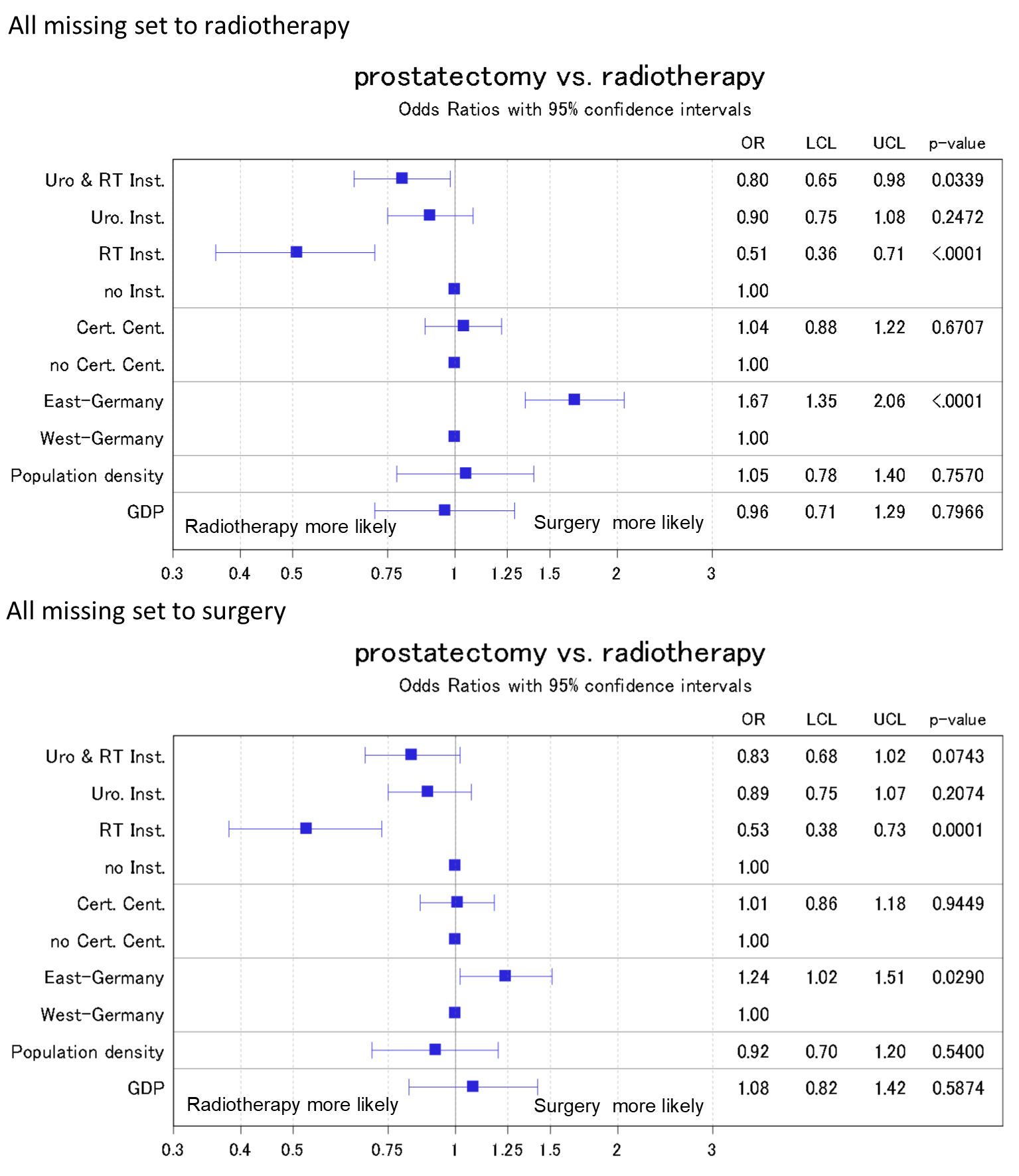

Supplement: Supplementary file 3 — Figure S3: Odds ratio from logistic regression models of the association of public health parameters with the administered treatment for the comparison of prostatectomy with surgery. Sensitivity analysis where all missing values were presumed to have been treated with surgery or radiotherapy, respectively. Models were adjusted for patient characteristics (age, age squared, probability of treatment in relation to age, grading and TNM-stage) with inverse probability weighting to account for missing data. ‘Radiotherapy institutions’ refers to in- and outpatient institutions, ‘urologic institutions’ refers to inpatient units only. (TIFF 437 kb) [file 432_2020_3140_MOESM3_ESM.tif]

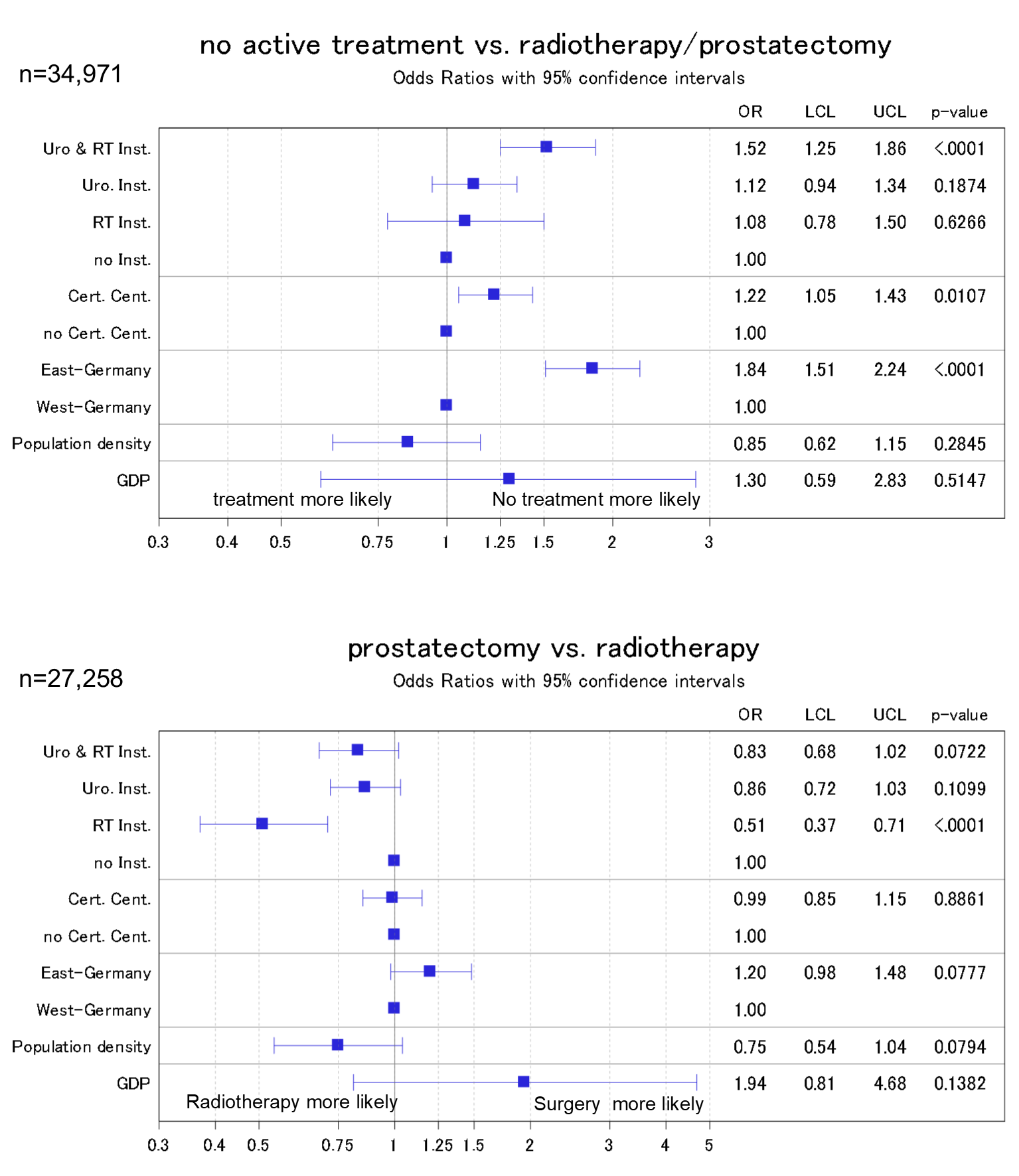

Supplement: Supplementary file 4 — Figure S4: Odds ratio from logistic regression models of the association of public health parameters with the administered treatment. Sensitivity analysis where cases from Saxony-Anhalt and Berlin were excluded. Models were adjusted for patient characteristics (age, age squared, probability of treatment in relation to age, grading and TNM-stage) with inverse probability weighting to account for missing data. ‘Radiotherapy institutions’ refers to in- and outpatient institutions, ‘urologic institutions’ refers to inpatient units only. (TIFF 454 kb) [file 432_2020_3140_MOESM4_ESM.tif]
